# Full-Length Genome Sequence of a Chikungunya Virus Isolate from the 2017 Autochthonous Outbreak, Lazio Region, Italy

**DOI:** 10.1128/genomeA.01306-17

**Published:** 2017-12-07

**Authors:** Fabrizio Carletti, Patrizia Marsella, Francesca Colavita, Silvia Meschi, Eleonora Lalle, Licia Bordi, Domenico Di Lallo, Vincenzo Panella, Antonino Di Caro, Emanuele Nicastri, Paola Scognamiglio, Simone Lanini, Francesco Vairo, Maria R. Capobianchi, Giuseppe Ippolito, Concetta Castilletti

**Affiliations:** a National Institute for Infectious Diseases L. Spallanzani, Rome, Italy; b Regional Health and Social Policy Department, Lazio Region, Rome, Italy; c Regional Service for Surveillance and Control of Infectious Diseases, Rome, Italy

## Abstract

We report here the genome sequence of a human chikungunya virus isolate from the ongoing autochthonous outbreak in central Italy. The sequence (East-Central-South African lineage, Indian Ocean sublineage), which is similar to recent sequences from Pakistan and India, shows E1 and E2 signatures of strains whose main mosquito vector is *Aedes aegypti*, although *Aedes albopictus* is the vector in Italy.

## GENOME ANNOUNCEMENT

Chikungunya virus (CHIKV) is an enveloped virus of the genus *Alphavirus*, family *Togaviridae*, with a single-stranded positive-sense RNA genome about 12,000 nucleotides (nt) long (1–3). CHIKV is widely distributed in tropical regions of Africa, Southeast Asia, the Indian subcontinent, the Pacific region, and, since 2013, the Americas. In European countries, autochthonous outbreaks were reported in 2007 (Emilia Romagna region, Italy) and in 2010, 2014, and 2017 (France) (4, 5). For the second time, Italy is currently experiencing autochthonous cases in the Lazio region (cities of Anzio, Latina, and Rome) and in the Calabria region (city of Guardavalle Marina) (5, 6). Autochthonous transmission is estimated to have started in Anzio in early to mid-June 2017 or earlier. As of 6 October 2017, 242 cases in Lazio (148 confirmed) and 33 in Calabria (5 confirmed) are acknowledged by the national surveillance system; 7 cases (3 confirmed) epidemiologically linked to Lazio have been reported in other Italian (*n =* 5) or European (*n =* 2) regions. As with the 2007 outbreak, it is likely that in the present outbreak, the index case is a viremic traveler yet to be identified. The massive presence of a competent vector (*Aedes albopictus*) and the exceptionally hot summer season of 2017 likely favored the establishment of a natural host-vector cycle.

Partial characterization of the surface glycoprotein E1 from the first human cases and from infected mosquitoes in the Lazio region indicates that the virus belongs to the East-Central-South African (ECSA) lineage and lacks the A226V adaptive mutation reported to increase replication in *A. albopictus* (7–11).

We report here the near-complete genome sequence of the first isolate, CHIKV/ITA/Lazio-INMI1-2017, obtained on BHK-21 cells from an acute-phase serum sample collected on 11 September 2017 from a patient living in Anzio. An 11,604-nt-long sequence was amplified in 23 overlapping reverse transcriptase-PCR amplicons and Sanger sequenced.

In the maximum-likelihood phylogenetic tree, CHIKV/ITA/Lazio-INMI1-2017 belongs to the Indian Ocean sublineage of ECSA. Unlike the strain causing the 2007 outbreak in Italy, the current strain clusters with recent Indian and Pakistan isolates, as well as with isolates obtained in China from travelers returning from India (12). Nucleotide similarity with the sequence of isolate Pakistan 07/2016 is >99% (21 mismatches over 11,604 nt).

Focusing on the main genetic signatures described for the E1 and E2 envelope glycoproteins, CHIKV/ITA/Lazio-INMI1-2017 shows the following polymorphisms compared to the reference S27 strain: K201E, M269V, D284E, I317V, and V322A in E1 and A164T, T312M, V264A, S375T, and V386A in E2. Considering the reported role of E1 and E2 polymorphisms in determining the fitness of CHIKV for the mosquito vector, it seems that, given the genetic signature of CHIKV/ITA/Lazio-INMI1-2017, *Aedes aegypti* is the preferred vector (7–9, 12–15). This may have implications for transmission efficiency in the present outbreak, given that *A. albopictus* is the unique competent vector, with *A. aegypti* not circulating in Italy.

Further characterization of the strain involved in the present outbreak, as well as assessment of possible microevolutionary trends, will be crucial for clarifying the infection dynamics and investigating the relevance of genetic signatures for virus fitness in local mosquito vectors and the extent of transmission in humans.

### Accession number(s).

The assembled complete genome sequence of chikungunya virus isolate CHIKV/ITA/Lazio-INMI1-2017 has been deposited in GenBank under the accession number MG049915.
